# Mental Health and Personality Functioning of People With Probable Personality Disorder Who Have Coexisting Complex Post Traumatic Stress Disorder

**DOI:** 10.1002/pmh.70010

**Published:** 2025-02-20

**Authors:** Sapphira McBride, Nia Goulden, Kirsten Barnicot, Kieron Corrigan, Sophie Shen, Serena Guillemard, Violet Effiom, Gemma Harrison, Lizwi Nyathi, Lyn Charles, Snehal P. Pandya, Verity Leeson, Rachel Evans, Mike J. Crawford

**Affiliations:** ^1^ Division of Psychiatry Imperial College London London UK; ^2^ North Wales Organisation for Randomised Trials in Health Bangor University Bangor Gwynedd UK; ^3^ School of Health & Medical Sciences St George's, University of London London UK; ^4^ Avon and Wiltshire Mental Health Partnership NHS Trust, Research and Development Blackberry Hill Hospital Bristol UK; ^5^ Research and Development Mersey Care NHS Foundation Trust, Hollins Park Warrington UK; ^6^ Department of Psychiatry University of Oxford Headington UK; ^7^ Research Department Coventry and Warwickshire Partnership Trust (CWPT) Coventry UK; ^8^ Centre for Research and Development, Derbyshire Healthcare NHS Foundation Trust Kingsway Hospital Derbyshire UK; ^9^ Clinical Research Office Lincolnshire Partnership NHS Foundation Trust Lincoln UK

**Keywords:** borderline personality disorder, comorbidity, complex post‐traumatic stress disorder, personality disorder, trauma

## Abstract

This paper examines the prevalence and comorbidity of complex post‐traumatic stress disorder (CPTSD) and borderline personality disorder (BPD) among individuals with probable personality disorder, using baseline data from the Structured Psychological Support clinical trial. The clinical characteristics and personality functioning of participants are summarised and compared between those meeting criteria for BPD, CPTSD, both or neither condition. Among 292 participants, 97% reported significant trauma exposure, and over half met the criteria for CPTSD. Those with CPTSD exhibited higher levels of social dysfunction and depression compared with those with BPD, despite both groups showing elevated emotion dysregulation and anxiety. Comorbidity of CPTSD and BPD was high, with 50% of the sample meeting criteria for both conditions. Participants with comorbid CPTSD and BPD displayed poorer baseline scores across all measures of mental health and functioning than those who met criteria for BPD alone. No statistically significant differences were found in suicidal behaviour or treatment‐seeking between groups. There were no significant differences in International Classification of Diseases‐11 personality trait domains between participants with CPTSD and BPD, but people with comorbid CPTSD and BPD displayed higher levels of trait negative affectivity than those with BPD alone. The findings highlight the need for trauma‐informed assessments in clinical settings and a better understanding of the impact of CPTSD on treatment outcomes for people with personality disorder, including how existing treatments may need to be modified to better meet the needs of people with these highly comorbid conditions.

**Trial Registration:**

Current controlled trials ISRCTN13918289 (registered 11/11/2022)

## 1 Background

The role of trauma in mental health has been extensively studied, demonstrating a strong, cumulative relationship between traumatic experiences and psychiatric morbidity (Karam et al. 2014; Kessler et al. 2010; Mauritz et al. 2013; McCrone et al. 2008; McKay et al. 2022; Schilling, Aseltine, and Gore 2007). Lifetime trauma is associated with poorer mental, physical and emotional health outcomes alongside pervasive economic and social costs, cementing trauma as a global public health issue (Felitti et al. 1998; Magruder, McLaughlin, and Elmore Borbon 2017; Mauritz et al. 2013; McCrone et al. 2008).

A range of personality difficulties are significantly linked to childhood adversity and trauma (Bierer et al. 2003; Yen et al. 2002). Borderline personality disorder (BPD) in particular has received a lot of attention for its consistent and strong associations with traumatic experiences in both childhood and adulthood (Herman, Perry, and van der Kolk 1989; Munjiza, Britvic, and Crawford 2019). People diagnosed with BPD are over 13 times more likely to report childhood adversity than non‐clinical controls and 3.5 times more likely to report childhood adversity than other psychiatric groups, with emotional abuse and neglect being especially prevalent (Porter et al. 2020). Furthermore, there is considerable overlap in symptom profiles of those with BPD and post‐traumatic stress disorder (PTSD) alongside high rates of comorbidity (Ford and Courtois 2021).

The close link between personality difficulties and trauma has sparked controversy surrounding the validity, reliability and utility of the current classification system of personality disorders. This position sits among existing controversies surrounding the BPD diagnosis in particular, stemming from issues with its reliability and validity as a distinct diagnostic construct (Knefel, Tran, and Lueger‐Schuster 2016) and the high rates of comorbidity with other disorders (Shah and Zanarini 2018). It has been suggested that BPD should be considered a trauma‐related condition, with symptoms reflecting the long‐term impact of prolonged, repeated or multiple forms of trauma (Gunderson and Sabo 2001; Herman, Perry, and van der Kolk 1989). However, experiencing a traumatic stressor is not a prerequisite for diagnosis of BPD, and evidence‐based treatments for the disorder, such as Dialectal Behaviour Therapy and Systems Training for Emotional Predictability and Problem Solving, tend not to have a trauma‐specific focus (Linehan 1987; Blum et al. 2008).

To account for the longer‐term impacts of experiencing trauma on mental health, the World Health Organization (WHO) introduced complex PTSD (CPTSD) as a diagnosis in its 11th version of the International Classification of Diseases (ICD‐11), under the category of ‘Disorders specifically associated with stress’. The diagnosis of CPTSD requires that an individual meet the diagnostic threshold for PTSD (re‐experiencing of the trauma, avoidance of trauma reminders and hypervigilance to threat), alongside three additional symptom clusters representing ‘disturbances of self‐organisation’ (DSO): affect dysregulation, negative self‐concept and difficulties in forming and maintaining relationships. The DSO symptoms of CPTSD have a strong resemblance to many of the difficulties central to BPD, and available studies have demonstrated high rates of comorbidity between the two disorders as well as substantial co‐occurrence of symptom endorsement (Ford and Courtois 2021; Jowett, Karatzias, and Albert 2020; Landy et al. 2015; Saraiya et al. 2021).

Whilst experiences of trauma and levels of PTSD are elevated among people with a range of personality disorders (Markowitz et al. 2015; Mauritz et al. 2013; Pietrzak et al. 2011), research examining links between CPTSD and personality disorder to date has focussed exclusively on BPD. Much of the research that has examined the relationship between CPTSD and BPD has been conducted among people with severe conditions such as those admitted to hospitals or those treated by specialist personality disorder treatment services (Borroni et al. 2021; Morris et al. 2021; van Dijke, Hopman, and Ford 2018). A Danish study examining the overlap between CPTSD and other diagnoses among outpatients included only 13 people with BPD, of whom six met the criteria for CPTSD (Møller et al. 2020). A secondary analysis of data from an Australian clinical trial reported that 45% of 82 people with BPD also met the criteria for CPTSD (Cloitre et al. 2014).

The extant literature, whilst mixed in findings, supports some degree of distinction between CPTSD and BPD diagnoses, with authors proposing different underlying neurobiological and psychosocial developmental pathways to the overlapping symptom profiles (Blay et al. 2024; Ford and Courtois 2021). However, it remains clinically and theoretically important to further disambiguate the two disorders, with Ford and Courtois (2021) highlighting the need for more precise prevalence and comorbidity data from a wider range of trauma‐exposed samples, using standardised and validated measures of CPTSD, namely, the International Trauma Questionnaire (ITQ; Cloitre et al. 2018, 2019). Additionally, it is important that research widen its scope beyond examining BPD in isolation, as the link between trauma, CPTSD and other personality difficulties remains understudied. The introduction of five trait domains of personality disturbance in the ICD‐11 simplifies the classification of personality disorders whilst acknowledging the dimensional nature of personality difficulties that is supported by the growing evidence base (Mulder 2021). A better understanding of how these trait domains map onto CPTSD, BPD and their co‐occurrence may shed light on the underlying psychological processes and developmental pathways involved.

The Structured Psychological Support (SPS) study is a single‐blind randomised controlled trial examining the clinical and cost‐effectiveness of low‐intensity psychological support for people with probable personality disorder. The ITQ was included as part of the baseline assessment to develop a better understanding of the connections between CPTSD and personality disorder (Crawford et al. 2024). We set out to use baseline data from the trial to examine the prevalence of CPTSD among a treatment‐seeking sample of patients with a broad range of personality‐related mental health problems and to compare the demographic and clinical characteristics within the sample.

In this exploratory analysis of baseline data from the trial, we examine levels of trauma, PTSD, DSO and CPTSD among study participants and compare the clinical and service use profiles of those who meet criteria for BPD, CPTSD, neither or both conditions.

## 2 Methods

### 2.1 Study Design

The data used were collected at baseline from the SPS study: a multicentre, randomised, parallel‐group, researcher‐masked, superiority trial investigating the clinical and cost‐effectiveness of a low‐intensity psychological treatment for individuals with personality disorder, the protocol for which has been published elsewhere (Crawford et al. 2024).

### 2.2 Recruitment Procedures

Study participants (*N* = 336) were recruited from primary and secondary care mental health services across seven National Health Service (NHS) sites in the Northwest, Southwest, Midlands, East and Southeast of England. Potential participants were invited to take part in the study if they were aged 18 or over, were being treated by mental health staff working in primary or secondary care services and were presenting to services with personality‐related difficulties. Potential participants were excluded if they were unable or unwilling to provide written informed consent; were receiving, or on a waiting list to receive, psychological treatment for personality disorder within the next 12 months; had a coexisting organic or psychotic mental disorder; or did not score four or higher on the Standardised Assessment of Personality Disorder–Abbreviated Scale (SAPAS), which indicates probable personality disorder (Moran et al. 2003). Clinicians at each study site were encouraged to refer potential participants if they had a formal diagnosis of personality disorder or had indications at assessment of personality‐related problems such as emotional distress and interpersonal difficulties. Once potential participants provided verbal consent to be contacted by a researcher, they were given an overview of the study rationale and procedures and provided with a Participant Information Sheet. All potential participants were given at least 24 h to consider taking part in the SPS study before they were invited to sign an Informed Consent Form. A researcher on the study then assessed each participant for eligibility via self‐report screening scales and a clinical records check. If eligible, participants completed a baseline assessment prior to randomisation and were given a £10 honorarium. All baseline data were collected between February 2023 and February 2024.

### 2.3 Study Measures

#### 2.3.1 SCID‐II

We used the Structured Clinical Interview for Axis II Personality Disorders (SCID‐II; First and Gibbon 2004) to determine whether participants met criteria for BPD. The SCID‐II is a semi‐structured diagnostic screening questionnaire and interview measure designed to offer a fast, reliable and valid clinical assessment of personality disorder. A score of three on five or more items fulfils the criteria for BPD. The SCID‐II demonstrates strong internal consistency and inter‐rater reliability (Jacobsberg, Perry, and Frances 1995; Lobbestael, Leurgans, and Arntz 2011; Maffei et al. 1997) and has been supported as a diagnostic tool for clinical and research purposes (Dreessen and Arntz 1998).

#### 2.3.2 ITQ

We used the ITQ (Cloitre et al. 2018) to assess symptoms of PTSD and CPTSD. We introduced the ITQ to study participants by explaining that we wanted to find out whether they had any traumatic experiences in the past and, if so, how these may have affected them. We gave study participants the option of not completing the ITQ.

The ITQ is a validated self‐report diagnostic measure that was developed to capture symptoms of PTSD and CPTSD in line with the WHO's ICD‐11 specifications. Participants are first asked whether they have had a previous traumatic experience and to briefly describe it, including when the trauma occurred. In keeping with the ICD‐11 definition of PTSD, we explained to participants that traumatic experiences could be single events or a series of events, such as a road traffic accident, being in a war zone, acts of violence, or sexual assault that you experience, or you see someone else experiencing. In cases where participants reported more than one traumatic experience, they were asked to identify a primary trauma for the purpose of completing the ITQ.

If the gateway criterion is fulfilled, participants answered six items representing the three PTSD symptom clusters (re‐experiencing, avoidance and sense of threat) and six items representing the three DSO symptom clusters (affective dysregulation, negative self‐concept and disturbed relationships). Participants were asked to rate how often they have been bothered by each symptom in the past month for PTSD items and to what extent each symptom typically applies to them for CPTSD items, using a 5‐point Likert scale ranging from 0 (*Not at all*) to 4 (*Extremely*). To fulfil ICD‐11 criteria for each disorder, participants were additionally asked three items capturing the level of functional impairment in the past month for each set of symptoms using the same 5‐point Likert scale. The measure is scored such that participants may fulfil criteria for PTSD or CPTSD, but not both.

The ITQ demonstrates strong psychometric properties, including internal consistency (Camden et al. 2023; Ho et al. 2019; Murphy et al. 2020), convergent and discriminant validity (Camden et al. 2023; Hyland et al. 2017) and good agreement with clinician‐administered diagnostic interviews (Hansen et al. 2021).

#### 2.3.3 PAQ‐11

We applied the Personality Assessment Questionnaire for ICD‐11 Personality Trait Domains (PAQ‐11; Kim, Tyrer, and Hwang 2021) to assess the five trait domains of personality disturbance specified by the ICD‐11: anankastia, detachment, disinhibition, dissociality and negative affectivity. The PAQ‐11 offers a short self‐report measure of the dimensional trait domain model of personality disorder introduced in the ICD‐11. Participants answer 17 items on a 5‐point Likert scale ranging from 0 (*Never*) to 4 (*Always*). The PAQ‐11 demonstrates acceptable internal consistency and adequate convergent and discriminant validity (Kim, Tyrer, and Hwang 2021). The total subscale scores can be used as a continuous measure of personality difficulty in each domain. Preliminary work to establish possible cut‐off scores for the presence of personality difficulties has also been conducted (Kim, Choi, and Tyrer 2024).

### 2.4 Other Measures

We applied the Work and Social Adjustment Scale (WSAS) to assess social and occupational functioning (Thandi, Fear, and Chalder 2017). We used the 16‐item Difficulties in Emotion Regulation Scale (DERS‐16; Bjureberg et al. 2016), the 9‐item Patient Health Questionnaire (PHQ‐9; Kroenke, Spitzer, and Williams 2001) and the 7‐item Generalised Anxiety Disorder Scale (Gad‐7; Spitzer et al. 2006) to assess mental health and items from the National Household Survey of Psychiatric Morbidity to assess suicidal behaviour (Thomas et al. 2002).

The incidence of attendance at Accident and Emergency services was recorded as part of an adapted version of the Adult Service Use Schedule (AD‐SUS; Borschmann et al. 2013; Crawford et al. 2018).

The baseline and screening measures also captured demographic details, including age, ethnicity, gender, median years of contact with health services, number and percentage married or in civil partnership and employment status.

### 2.5 Data Analysis

We calculated the proportion of people in the sample who did and did not meet diagnostic criteria for BPD, ICD‐11 trait domains and CPTSD, with 95% confidence intervals. Study participants were then divided into four groups according to whether they met criteria for BPD only, CPTSD only, BPD and CPTSD or neither condition. We used multilevel mixed effects linear regression to compare scores on continuous measures of mental health, social functioning and ICD‐11 personality trait domains between these groups. Fixed factors included in the model were BPD (Yes/No, from SCID‐II at screening), CPTSD (Yes/No, from ITQ at screening) and a BPD * CPTSD interaction. Stratification variables were also included: site as a random effect and self‐identified gender (female/male/non‐binary or other) as a fixed effect. For binary variables, e.g., accident and emergency attendance during the previous 6 months, and for an exploratory analysis using the suggested cut‐offs for presence versus absence of ICD‐11 personality trait domain difficulties (Kim, Choi, and Tyrer 2024), we used multilevel mixed‐effects logistic regression. The same fixed and random factors were included in the multilevel mixed‐effects linear regression models detailed above. Comparisons between groups were corrected for multiple comparisons using Bonferroni correction. All data were analysed using STATA Version 18.

## 3 Results

Between 7 February 2023 and 31 January 2024, 336 people were recruited to the SPS trial. Most trial participants identified as female (*n* = 251, 74.7%), of White British ethnicity (*n* = 258, 76.8%) and their average age was 35 years (SD = 13.24). A total of 247 participants (73.5%, 95% CI = 68.2%–78.6%) met criteria for BPD according to the SCID‐II. Scores on the PAQ‐11 indicated that 252 (75.0%, 95% CI = 70.0%–79.5%) met ICD‐11 criteria for disinhibition, 251 (74.7%, 95% CI 70.0%–79.5%) for negative affectivity, 204 (61.5%, 95% CI = 56.0%–66.7%) for detachment, 145 (43.2%, 95% CI = 37.8%–48.6%) for anankastia and 117 (34.8%, 95% CI = 29.7%–40.2%) for dissociality.

Of the 336 people taking part in the SPS trial, 292 (86.9%) completed the ITQ, and 44 (13.1%) declined. Demographic and clinical characteristics of the sample for this analysis and the 44 non‐participants are presented in Table 1. The 292 people who completed the ITQ provide the sample for the rest of the results presented in this paper. Among this group, 215 (73.6%) met criteria for BPD, and 283 (96.9%) reported having experienced significant trauma in their lives. A total of 190 participants (65.1%, 95% CI [0.59, 0.71]) met criteria for PTSD, 250 (85.6%, 95% CI [0.81, 0.89]) met criteria for DSO and 181 (62.0%, 95% CI [0.56, 0.68]) met criteria for CPTSD. Half of all participants (*n* = 145, 50.0%) met criteria for both BPD and CPTSD.

**TABLE 1:** Demographic and clinical characteristics of participants who did and did not complete the International Trauma Questionnaire (ITQ).

Characteristic	Descriptive	Completed the ITQ	Did not complete the ITQ	Total
Age	*N*	292	44	336
	Mean (SD)	35.20 (13.64)	32.18 (9.99)	34.81 (13.24)
Gender (identified)	Male *N* (%)	64 (21.9)	11 (25)	75 (22.3)
	Female *N* (%)	218 (74.7)	33 (75)	251 (74.7)
	Nonbinary/other *N* (%)	10 (3.4)	0 (0)	10 (3.0)
Years of contact with health service	*N*	291	44	335
	Median [IQR]	10 [6–20]	10 [4–21]	10 [6–20]
Married or in civil partnership	*N* (%)	35 (11.99)	6 (13.64)	41 (12.2)
Months in full time employment from previous 6 months	*N*	291	44	335
	Median [IQR]	0 [0–6]	0 [0–6]	0 [0–6]
Total WSAS score	*N*	289	44	333
	Mean (SD)	30.05 (6.40)	28.61 (6.35)	29.86 (6.40)
Total DERS score	*N*	287	44	331
	Mean (SD)	65.71 (9.84)	63.71 (10.75)	65.45 (9.97)
Total PHQ score	*N*	291	44	335
	Mean (SD)	19.36 (4.69)	17.52 (5.31)	19.12 (4.81)
Total GAD‐7 score	*N*	291	44	335
	Mean (SD)	15.67 (4.14)	13.39 (4.36)	15.37 (4.24)
Life not worth living	*N* (%)	264 (90.41)	39 (88.64)	303 (90.18)
Deliberate self‐harm	*N* (%)	132 (45.21)	19 (43.18)	151 (44.94)
A&E attendance	*N* (%)	116 (39.73)	10 (22.73)	126 (37.50)
Ethnicity				
White—British	*N* (%)	225 (77.05)	33 (75.00)	258 (76.79)
White—Irish	*N* (%)	3 (1.03)	0 (0)	3 (0.89)
White—Other	*N* (%)	17 (5.82)	3 (6.82)	20 (5.95)
Mixed—White and Black Caribbean	*N* (%)	3 (1.03)	2 (4.55)	5 (1.49)
Mixed—White and Asian	*N* (%)	2 (0.68)	1 (2.27)	3 (0.89)
Mixed—Other mixed	*N* (%)	10 (3.42)	1 (2.27)	11 (3.27)
Asian or Asian British—Indian	*N* (%)	5 (1.71)	0 (0)	5 (1.49)
Asian or Asian British—Pakistani	*N* (%)	4 (1.37)	2 (4.55)	6 (1.79)
Asian or Asian British—Bangladeshi	*N* (%)	1 (0.34)	0 (0)	1 (0.30)
Asian or Asian British—Other Asian	*N* (%)	4 (1.37)	0 (0)	4 (1.19)
British or Black British—Caribbean	*N* (%)	11 (3.77)	1 (2.27)	12 (3.57)
British or Black British—African	*N* (%)	4 (1.37)	0 (0)	4 (1.19)
British or Black British—Other Black	*N* (%)	1 (0.34)	0 (0)	1 (0.30)
Other ethnic group	*N* (%)	2 (0.68)	1 (2.27)	3 (0.89)

Adjusted mean scores on measures of mental health and social functioning are presented in Table 2 according to whether participants met criteria for BPD only, CPTSD only, both BPD and CPTSD or neither condition. Assumptions of the analysis models were checked and all deemed to hold. Levels of mental distress and social dysfunction were high across all study groups and higher among people with CPTSD alone compared with people with BPD. All measures of mental distress and social dysfunction were statistically significantly higher among those with both CPTSD and BPD than they were among people with only BPD or those who did not meet the threshold for BPD. The WSAS scores were statistically significantly higher among those with both BPD and CPTSD compared with those with BPD only (3.95, *p* < 0.001), and among those with CPTSD only compared with BPD only (4.21, *p* < 0.005), indicating poorer social functioning. The DERS‐16 scores were statistically significantly higher among those with both BPD and CPTSD compared with those with BPD only (6.65, *p* < 0.001), indicating more difficulty with emotion regulation, and statistically significantly lower among those without BPD or CPTSD compared with those with BPD only (−5.64, *p* = 0.007), indicating less difficulty with emotion regulation. The PHQ‐9 scores were statistically significantly higher among those with both BPD and CPTSD compared with those with BPD only (3.12, *p* < 0.001), and among those with CPTSD only compared with BPD only (2.52, *p* = 0.034), indicating more symptoms of depression. The GAD‐7 scores were statistically significantly higher among those with both BPD and CPTSD compared with those with BPD only (3.16, *p* < 0.001), indicating more symptoms of anxiety. Clinical interpretation of mean scores on the PHQ‐9 and GAD‐7 indicates that those with CPTSD and BPD displayed severe levels of depression and anxiety; those with BPD alone or with personality difficulties that did not meet the threshold for BPD displayed moderately severe depression and moderate anxiety; and those with CPTSD alone displayed severe depression and moderate anxiety.

**TABLE 2:** Mean differences in mental health and social functioning among study participants.

Outcome measure	BPD and CPTSD status—*N*, adjusted mean (95% CI)				Difference in means between groups (95% CI)		
	BPD only	CPTSD only	Both BPD and CPTSD	Neither BPD nor CPTSD	CPTSD vs. BPD	CPTSD and BPD vs. BPD	No BPD/CPTSD vs. BPD
Social dysfunction (WSAS)	*N* = 67, 27.68 (26.15–29.21)	*N* = 36, 31.88 (29.85–33.92)	*N* = 39, 31.63 (30.54–32.72)	*N* = 144, 26.08 (24.13–28.02)	4.21 (0.88–7.53) *p* = 0.005*	3.95 (1.59–6.31) *p* < 0.001**	−1.60 (−4.79 to 1.59) *p* = 1.000
Difficulties in emotional regulation (DERS‐16)	*N* = 68, 62.70 (26.15–29.21)	*N* = 36, 65.69 (29.85–33.9)	*N* = 39, 69.35 (30.54–32.72)	*N* = 141, 57.06 (24.13–28.02)	2.99 (−1.79 to 7.77) *p* = 0.594	6.65 (3.25–10.05) *p* < 0.001**	−5.64 (−10.25 to −1.03) *p* = 0.007*
Depressive symptoms (PHQ‐9)	*N* = 68, 17.57 (16.44–18.70)	*N* = 36, 20.09 (18.59–21.59)	*N* = 39, 20.69 (19.86–21.52)	*N* = 145, 16.21 (14.78–17.65)	2.52 (0.12–4.91) *p* = 0.034*	3.12 (1.43–4.81) *p* < 0.001**	−1.36 (−3.66 to 0.94) *p* = 0.717
Symptoms of anxiety (GAD‐7)	*N* = 68, 13.98 (13.00–14.97)	*N* = 36, 14.81 (13.50–16.13)	*N* = 39, 17.14 (16.43–17.86)	*N* = 145, 13.77 (12.51–15.03)	0.83 (−1.82 to 2.25) *p* = 1.000	3.16 (1.66–4.66) *p* < 0.001**	−0.21 (−2.25 to 1.82) *p* = 1.000

*p < 0.05, **p < 0.001.

The proportion of people reporting suicidal behaviour, attending emergency departments and calling mental health crisis lines is presented in Table 3. There were no statistically significant differences between study groups.

**TABLE 3:** Differences in suicidal behaviour and service use in previous 6 months between study groups.

Outcome measure	BPD and CPTSD status—adjusted proportion (95% CI)				Difference in log odds (95% CI) between groups		
	BPD only	CPTSD only	Both BPD and CPTSD	Neither BPD nor CPTSD	CPTSD vs. BPD	CPTSD and BPD vs. BPD	No BPD/CPTSD vs. BPD
	*N* = 69	*N* = 36	*N* = 39	*N* = 145			
Proportion reporting life is not worth living	0.90 (0.83–0.98)	0.87 (0.73–1.00)	0.95 (0.91–0.99)	0.72 (0.55–0.89)	−0.39 (−2.29 to 1.51) *p* = 1.000	0.73 (−0.79 to 2.25) *p* = 1.000	−1.38 (−2.97 to 0.20) *p* = 0.127
Proportion reporting having self‐harmed	0.48 (0.35–0.60)	0.34 (0.18–0.50)	0.52 (0.43–0.61)	0.23 (0.10–0.37)	−0.57 (−1.74 to 0.59) *p* = 1.000	0.18 (−0.6 to 10.97) *p* = 1.000	−1.10 (−2.3 to 10.11) *p* = 0.097
Proportion attending emergency department	0.43 (0.30–0.57)	0.32 (0.26–0.49)	0.42 (0.33–0.52)	0.32 (0.16–0.47)	−0.49 (−1.70 to 0.72) *p* = 1.000	−0.04 (−0.86 to 0.78) *p* = 1.000	−0.51 (−1.68 to 0.65) *p* = 1.000
Proportion calling a mental health crisis line	0.27 (0.14–0.40)	0.38 (0.20–0.56)	0.40 (0.28–0.51)	0.31 (0.15–0.47)	0.57 (−0.68 to 1.82) *p* = 1.000	0.63 (−0.27 to 1.54) *p* = 0.393	0.23 (−0.99 to 1.45) *p* = 1.000

*p < 0.05, **p < 0.001.

Levels of ICD‐11 personality trait domains among study groups based on linear and logistic models are presented in Tables 4 and 5, respectively. The highest scoring trait domain among people with BPD only was disinhibition; the highest scoring trait domain among people with CPTSD only was negative affectivity; and among people with both conditions, both disinhibition and negative affectivity were the dominant traits displayed. However, there were no statistically significant differences between participants with BPD and CPTSD only on any trait domains. Levels of negative affectivity were statistically significantly higher among those with both BPD and CPTSD compared with those with BPD only based on the linear model (mean difference = 1.45, 95% CI = 0.36–2.55, *p* = 0.003), although there was no statistically significant difference in the proportions scoring above the preliminary cut‐off score for this domain. Levels of disinhibition were statistically significantly higher among those with BPD compared with those with neither BPD nor CPTSD, based on both the linear model and the proportions scoring above the cut‐off score for this domain (mean difference −0.89, 95% CI = −1.74 to 0.04, *p* = 0.035; difference in log odds −1.21, 95% CI = −2.40 to −0.02, *p* = 0.044).

**TABLE 4:** Mean differences of trait domains from PAQ‐11, split by diagnosis group.

Trait domains	BPD and CPTSD status—adjusted mean (95% CI)				Difference in means between groups (95% CI)		
	BPD only	CPTSD only	Both BPD and CPTSD	Neither BPD nor CPTSD	CPTSD vs. BPD	CPTSD and BPD vs. BPD	No BPD/CPTSD vs. BPD
	*N* = 69	*N* = 36	*N* = 145	*N* = 39			
Negative affectivity	15.64 (14.96–16.33)	16.70 (15.77–17.64)	17.10 (16.62–17.57)	14.89 (13.99–15.80)	1.06 (−0.49 to 2.61) *p* = 0.422	1.45 (0.36–2.55) *p* = 0.003*	−0.75 (−2.26 to 0.76) *p* = 1.000
Detachment	8.90 (8.16–9.65)	9.27 (8.32–10.22)	9.75 (9.15–10.34)	8.40 (7.48–9.32)	0.37 (−1.04 to 1.78) *p* = 1.000	0.84 (−0.15 to 1.83) *p* = 0.146	−0.51 (−1.86 to 0.85) *p* = 1.000
Dissociality	2.17 (1.73–2.61)	1.56 (0.99–2.12)	2.26 (1.91–2.60)	1.80 (1.25–2.34)	−0.62 (−1.48 to 0.24) *p* = 0.346	0.08 (−0.52 to 0.68) *p* = 1.000	−0.38 (−1.20 to 0.44) *p* = 1.000
Disinhibition	5.57 (5.19–5.95)	4.80 (4.27–5.32)	6.02 (5.76–6.28)	4.68 (4.18–5.19)	−0.96 (−2.18 to 0.26) *p* = 0.228	0.45 (−0.17 to 1.08) *p* = 0.338	−0.89 (−1.74 to −0.04) *p* = 0.035*
Anankastia	9.51 (8.51–10.51)	9.50 (8.20–10.81)	9.77 (9.00–10.53)	8.28 (7.03–9.53)	−0.01 (−2.03 to 2.01) *p* = 1.000	0.25 (−1.17 to 1.67) *p* = 1.000	−1.24 (−3.17 to 0.70) *p* = 0.550

*p < 0.05, **p < 0.001.

**TABLE 5:** Proportion of participants with different trait domains from PAQ‐11, split by diagnosis group.

Trait domains	BPD and CPTSD status—adjusted proportion (95% CI)				Difference in log odds (95% CI) between groups		
	BPD only	CPTSD only	Both BPD and CPTSD	Neither BPD nor CPTSD	CPTSD vs. BPD	CPTSD and BPD vs. BPD	No BPD/CPTSD vs. BPD
	*N* = 69	*N* = 36	*N* = 145	*N* = 39			
Negative affectivity	0.67 (0.56–0.79)	0.81 (0.67–0.94)	0.82 (0.76–0.89)	0.58 (0.42–0.74)	0.70 (−0.62 to 2.03) *p* = 0.959	0.80 (−0.10 to 1.70) *p* = 0.111	−0.39 (−1.51 to 0.73) *p* = 0.920
Detachment	0.54 (0.41–0.67)	0.69 (0.52–0.86)	0.70 (0.62–0.79)	0.52 (0.35–0.68)	0.66 (−0.58 to 1.91) *p* = 0.949	0.75 (−0.09 to 1.59) *p* = 0.112	−0.09 (−1.02 to 1.21) *p* = 1.000
Dissociality	0.36 (0.23–0.49)	0.29 (0.13–0.44)	0.38 (0.29–0.48)	0.39 (0.23–0.56)	−0.34 (−1.57 to 0.89) *p* = 1.000	0.09 (−0.74 to 0.93) *p* = 1.000	0.14 (−0.99 to 1.28) *p* = 1.000
Disinhibition	0.79 (0.69–0.89)	0.59 (0.43–0.75)	0.85 (0.79–0.91)	0.53 (0.37–0.69)	−0.96 (−2.18 to 0.26) *p* = 0.228	0.43 (−0.61 to 1.47) *p* = 1.000	−1.21 (−2.40 to −0.02) *p* = 0.044*
Anankastia	0.43 (0.29–0.57)	0.41 (0.23–0.58)	0.46 (0.34–0.57)	0.36 (0.20–0.53)	−0.10 (−1.27 to 1.06) *p* = 1.000	0.11 (−0.71 to 0.93) *p* = 1.000	−0.29 (−1.41 to 0.84) *p* = 1.000

*p < 0.05, **p < 0.001.

## 4 Discussion

In this secondary exploratory analysis of baseline data from the SPS trial, we provide new insight on the prevalence of CPTSD among people who are in contact with secondary mental health services and have probable personality disorder. Nearly all those who took part in the trial and completed the ITQ reported having experienced one or more significant traumas in their lives, and over half met criteria for CPTSD. Levels of mental distress were high across the sample, and study participants displayed a mean of 29.9 on the WSAS, much higher than the threshold of 20, indicating moderate/severe impairment. Participants who met the criteria for CPTSD displayed similarly high levels of emotion dysregulation and anxiety as those with BPD and even higher levels of social dysfunction and depression. Participants with comorbid BPD and CPTSD scored higher on all measures of social dysfunction, emotion dysregulation, depression and anxiety and displayed statistically significantly higher levels of ICD‐11 trait negative affectivity than those with BPD alone. We found statistically significant differences indicating that participants who met the diagnostic threshold for BPD displayed higher levels of emotion dysregulation and ICD‐11 trait disinhibition than participants who did not meet criteria for either BPD or CPTSD, and these were the only constructs found to differentiate the diagnostic groups. There were no statistically significant differences between participants with BPD and CPTSD only on any personality trait domains.

Comorbidity of CPTSD with BPD in the study sample was high, with half of all participants meeting criteria for both conditions. Previous studies have also noted high levels of comorbidity between CPTSD and BPD (Atkinson et al. 2024; Ford and Courtois 2021). The high levels of overall trauma reported in the current study support the view that most patients presenting to mental health services with personality‐related difficulties have experienced significant trauma, despite trauma histories not being part of routine assessment in the diagnosis of personality disorders or used to inform the treatment approach (Barnicot and Crawford 2018).

The results of this study add evidence to the extant literature about similarities and differences between CPTSD and BPD. Whilst people with CPTSD and those with BPD shared high levels of emotion dysregulation and anxiety, people with CPTSD, either alone or in combination with BPD, displayed even higher levels of social dysfunction and depression than those with BPD. The results of several studies have suggested that BPD can be differentiated from CPTSD on the basis of greater impulsivity and disinhibition, including of aggressive or violent outbursts, and greater suicidal or self‐injurious behaviour (Atkinson et al. 2024). However, we observed no statistically significant differences in levels of disinhibition or suicidal behaviour between those with BPD and CPTSD, suggesting that these constructs may not differentiate the two clinical diagnoses as strongly as previously argued. Differences in interpersonal difficulties have also been proposed to differentiate BPD from CPTSD, with the former diagnosis associated with unstable and intense relationships alongside efforts to avoid real or perceived abandonment, and the latter diagnosis more marked by significant avoidance and emotional detachment (Blay et al. 2024; Ford and Courtois 2021; Møller et al. 2021). Whilst a higher proportion of the CPTSD‐only group displayed detachment difficulties than the BPD‐only group on the PAQ‐11, this was not a statistically significant difference, suggesting that differences in interpersonal function between the two diagnoses, as well as in disinhibition and suicidal behaviour, may not be as clear‐cut as previously argued. This view aligns with the dimensional approach taken by the Hierarchical Taxonomy of Psychopathology (HiTOP) model, which acknowledges both externalising and internalising features (e.g., disinhibition and detachment, respectively) as central to BPD, and with studies that have found BPD subgroups emerge on the basis of different internalising–externalising profiles (Eaton et al. 2011; Gamache et al. 2021; James and Taylor 2008; Kotov et al. 2017, 2021).

The only personality trait domain found to be significantly higher among the comorbid group compared with the BPD‐only group was negative affectivity, which was similarly elevated among the CPTSD‐only group. Given that negative affectivity captures feelings such as shame, guilt and low self‐esteem (Bach and First 2018), our results provide additional support for the idea that CPTSD is associated with more chronic negative self‐perceptions than BPD, whilst people with BPD may have a more unstable and fluctuating sense of self than those with CPTSD (Frost et al. 2020; Hyland et al. 2019; Saraiya et al. 2021). It is possible that negative affectivity, i.e., an increased tendency to experience negative emotions, poses a risk factor for developing CPTSD and BPD and/or acts as a pathway through which trauma impacts mental health in the form of overlapping CPTSD and BPD symptomatology. Longitudinal research investigating personality trait domains and subsequent psychological and clinical profiles would help to further clarify the role of negative affectivity, disinhibition and detachment in the development of BPD and CPTSD.

This study has a number of strengths. Our data help to answer previous calls for more studies investigating the prevalence and comorbidity of BPD and CPTSD (Ford and Courtois 2021) and widen the scope of existing literature in this area, which has predominantly focused on the overlap of BPD and CPTSD, to include a sample presenting with a range of personality disturbances, as captured by the PAQ‐11. Indeed, it is the first study to the author's knowledge to collect data on ICD‐11 trait domains of people with probable personality disorder who do and do not meet criteria for CPTSD. Several studies in this area have excluded participants who do not have an existing diagnosis of personality disorder, are taking psychotropic medications, or have coexisting difficulties such as substance misuse disorder (Møller et al. 2021; Saraiya et al. 2021; van Dijke, Hopman, and Ford 2018). The pragmatic design of the SPS trial, in which people with coexisting conditions (besides psychosis) were not excluded, increases the ecological validity of our results. Additionally, we deployed validated measures of both CPTSD and BPD, the use of which has been inconsistent across the existing literature (Atkinson et al. 2024).

However, the present study is also limited in the following ways. Firstly, data were missing from those participants who declined to complete the ITQ, which may have led us to under‐ or overestimate the prevalence of CPTSD within the sample. We did not capture reasons for participants declining to answer the ITQ. However, descriptive and clinical characteristics were similar among those who did and those who did not complete this measure. The results of the present study could have been enriched by using validated measures of the number and type of traumas experienced to test for differences between those with BPD, CPTSD, both or neither condition. Moreover, the clinical trial from which the present data were drawn was powered to examine the clinical effectiveness of SPS, a low‐intensity psychological intervention. Whilst there was sufficient power to identify differences in mental health over 12 months of follow‐up, the trial was not powered to look at the differences between groups for the current analysis (BPD only, CPTSD only, BPD and CPTSD and neither BPD nor CPTSD). The number of participants in certain groups means that there may not have been sufficient power to detect statistically significant differences. A larger study would be better powered to detect clinically important differences between these groups of patients in terms of service use and suicidal behaviour and would allow for a more detailed examination of ICD‐11 personality trait domains, potentially uncovering more pronounced differences between groups than were found in the current analysis. The statistical power of the logistic analysis of ICD‐11 personality trait domains may also be limited in detecting differences due to the dichotomisation of variables.

The results of this study highlight the need for further research to examine the complex relationship between trauma, personality disorder and CPTSD, with significant implications for clinical practice. Among our sample, there were many individuals who met criteria only for PTSD or CPTSD. Whilst we did not use diagnostic assessments of personality disorders other than BPD, our results suggest that there is a high prevalence of PTSD and CPTSD across the spectrum of personality‐related mental health disturbance. As such, clinicians should consider incorporating assessments like the ITQ for clients presenting with personality‐related distress, alongside considering implications for treatment planning.

In contrast to the extensive literature on the aetiology of BPD, which has identified interactions between a number of heritable and environmental factors (Paris 2023), less is known about the aetiology of CPTSD. Among people who have PTSD, those exposed to earlier onset of trauma and trauma that is more interpersonal in nature, e.g., familiarity with the perpetrator, appear to be more likely to develop CPTSD (Guzman Torres, Krause‐Utz, and Sack 2023). An earlier age of onset of trauma and familiarity with the perpetrator are also risk factors for the development of traits of BPD (Belsky et al. 2012). It is possible that future research into the aetiology of CPTSD will identify other factors that are shared with the aetiology of BPD and that both conditions will be seen as the product of interactions between environmental and heritable factors, in the same way that PTSD is increasingly understood (Amstadter et al. 2024).

There is an extensive evidence‐base for the effectiveness of psychological treatments for BPD, however, far less is currently known about how to best support people with CPTSD or its comorbidity with BPD. Due to the novelty of the diagnosis, there are limited trials of interventions with patients confirmed to meet diagnostic criteria for CPTSD. However, evidence from trials with patients likely to have CPTSD symptoms supports the benefit of trauma‐focused interventions (Karatzias et al. 2019; Mahoney, Karatzias, and Hutton 2019; Sele et al. 2023). There is preliminary evidence that it can be acceptable, safe and effective to combine evidence‐based interventions for BPD with those for PTSD in people with a dual diagnosis or for people with BPD who are childhood sexual abuse survivors; however, this approach has not been tested for people with BPD and confirmed CPTSD (Bohus et al. 2020; Harned, Korslund, and Linehan 2014; Harned et al. 2021; Kleindienst et al. 2021). Given the elevated levels of social dysfunction, depression and anxiety we found among individuals with both conditions, further development in this area is crucial. The SPS trial from which the current data were drawn will involve a secondary analysis of data to explore the impact of CPTSD on treatment outcomes, which will provide an important step in treatment development.

Participants in our study presented with high levels of a range of personality disturbances according to the ICD‐11 trait domains, and as such, future clinical trials of interventions for CPTSD and BPD would benefit from assessing how, and to what extent, dominant personality traits affect treatment outcomes. The next step may be to adapt interventions to target the difficulties associated with the dominant trait(s) identified, thereby adopting a more person‐centred approach to treatment. To facilitate this research, it would be helpful to investigate the relationship between the ICD‐11 trait domains as used in the present study and the DSM‐5 alternative model of personality disorder (AMPD), which also adopts a 5‐domain dimensional trait model, but with the domains of negative affectivity, detachment, antagonism, psychoticism and disinhibition (Widiger and Hines 2022). Criterion A of the DSM‐5's Alternative Model of Personality Disorders, levels of personality functioning, was specifically developed to help distinguish personality disorders from other types of mental health disorders. Future research comparing levels of impairment in self and interpersonal functioning may therefore help elucidate the relationship between CPTSD and BPD (Morey et al. 2022). Specifically, investigating more precisely the shared and distinct manifestations of maladaptive self and interpersonal functioning and how these contribute to comorbidity, functional impairment and treatment trajectories may shed light on the developmental pathways and underlying mechanisms of each disorder, helping to illuminate the relationship between CPTSD and BPD whilst addressing existing controversies surrounding their construct validity.

## 5 Conclusions

Our findings highlight significant overlap between BPD and CPTSD, particularly in terms of high levels of emotion dysregulation and anxiety, and suggest that coexisting CPTSD is associated with even higher levels of social dysfunction, depression and ICD‐11 trait negative affectivity. The high comorbidity between CPTSD and BPD reinforces the need for trauma‐informed assessments in clinical practice, particularly for clients presenting with personality‐related distress. Moreover, the high levels of ICD‐11 personality trait domains exhibited across groups point to the relevance of dimensional models of personality for understanding these disorders. Future research should focus on examining interactions between the role and type of trauma exposure, personality traits, heritable factors and the impact of these on treatment outcomes. Integrating these findings into personalised, trauma‐focussed and/or trait‐targeted interventions for comorbid CPTSD and BPD holds promise for improving clinical outcomes in this complex and underserved population.

## Ethics Statement

The SPS study was approved by the Health Research Authority (IRAS ID: 315951) prior to the start of data collection. The research was conducted in compliance with the Helsinki Declaration II and General Data Protection Regulations. Study findings will be published in an open‐access peer‐reviewed journal and disseminated at academic conferences.

## Consent

All participants were required to sign a consent form to participate and received a copy of their signed consent form prior to data collection.

## Conflicts of Interest

The authors declare no conflicts of interest.

## Data Availability

Data will be made available upon reasonable request following publication of the main trial findings.
